# Diagnostics of Bolted Joints in Vibrating Screens Based on a Multi-Body Dynamical Model

**DOI:** 10.3390/ma16175794

**Published:** 2023-08-24

**Authors:** Pavlo Krot, Hamid Shiri, Przemysław Dąbek, Radosław Zimroz

**Affiliations:** Faculty of Geoengineering, Mining and Geology, Wroclaw University of Science and Technology, Na Grobli 15, 50-421 Wroclaw, Poland; hamid.shiri@pwr.edu.pl (H.S.); przemyslaw.dabek@pwr.edu.pl (P.D.); radoslaw.zimroz@pwr.edu.pl (R.Z.)

**Keywords:** vibrating screen, bolted joints, clearances, nonlinear stiffness, dynamical model, modal analysis, resonance, diagnostics, damping, phase space plot

## Abstract

The condition-based maintenance of vibrating screens requires new methods of their elements’ diagnostics due to severe disturbances in measured signals from vibrators and falling pieces of material. The bolted joints of the sieving deck, when failed, require a lot of time and workforce for repair. In this research, the authors proposed the model-based diagnostic method based on modal analysis of the 2-DOF system, which accounts for the interaction of the screen body and the upper deck under conditions of bolted joint degradation. It is shown that the second natural mode with an out-of-phase motion of the upper deck against the main screen housing may coincide with the excitation frequency or its higher harmonics, which appear when vibrators’ bearings are in bad condition. This interaction speeds up bolt loosening and joint opening by the dynamical loading of higher amplitude. The proposed approach can be used to detune the system from resonance and anti-resonance to reduce maintenance costs and energy consumption. To prevent abrupt failures, such parameters as second natural mode frequency, damping factor, and phase space plot (PSP) distortion measures are proposed as bolt health indicators, and these are verified on the laboratory vibrating screen. Also, the robustness is tested by the impulsive non-Gaussian noise addition to the measurement data. A special diagram was proposed for the bolted joints’ strength capacity assessment and maintenance actions planning (tightening, replacement), depending on clearance in the joints.

## 1. Introduction

Different vibrating screens are widely used in raw material processing and aggregate industries. Vibration screens have a significant role in separating bulk materials such as the fractions of coal and ore. Sieving screens can separate from 10 to over 1000 tons per hour depending on their design, drive power, size, and the number of decks [1,2]. Cyclic excitation for screen deck motion can be produced by unbalanced rotating shafts, hydraulic cylinders, or electromagnetic actuators. Using decks and particle motion criteria, screens are classified into linear, elliptical, or circular types. Several decks can be utilized to increase general productivity and the final quality of the product.

### 1.1. Problems in Vibrating Sieving Screen Operation and Diagnostics

Sieving material is fed to the top of the screen by the belt conveyor with a specific linear speed. Some systems are under development [3] to prevent damage to equipment and improve process monitoring, but the conventional way is to design the upper deck of the screen as massive grizzly bars for providing scalping of the intake stream from the oversized components. The upper deck and lower levels of sieves are subjected to blinding, which induces screen overloading and technological process interruption. Also, large falling fragments of material generate force impacts with significant amplitude; thus, those disturbances which are stochastic by nature should be considered to prevent false alarms in fault diagnostic procedures [4,5,6,7,8]. The impulsive noise cancellation method for copper ore crusher vibration signal enhancement is proposed in [9]. The selection of the informative frequency band in a bearing fault diagnosis in the presence of non-Gaussian noise with a comparison of recently developed methods is fulfilled in [10]. Battery-powered wireless sensors are designed in [11] which are able to withstand vibratory loading and are embedded in the rubber screens for condition monitoring. Nevertheless, diagnostics of vibrating machines, in particular sieving screens, still is a challenge in theory and practice.

At the design stage, by using the discrete and finite element approach [12,13,14,15,16,17,18,19], the natural modes of the screen are examined to confirm minimal structural stresses and required trajectories of bulk material motion. Nonetheless, these approaches need significant computing resources in optimization and research. Also, statistical fraction distribution in the input flow, particle configuration, and a 3D screen model with details are required [20]. Hence, the reduced degree-of-freedom spring–mass models can be appropriate to accomplish a dynamical analysis of a vibrating screen as a set of rigid bodies connected by links with a certain stiffness and mass [21,22,23,24,25]. To account for the non-linear features of such systems, a piecewise model and estimates of damping and natural frequency are implemented in [26], including adaptive diagnosis of the bilinear mechanical systems based on the free oscillation method with the decrement as a recognition feature [27].

Supporting springs are well-known as vital components of sieving screens, and they substantially affect particles’ trajectory and overall process efficiency due to a significant influence on their dynamics [28]. Although there are a few advantages of using elastomeric or air-filled springs, they could create nonlinear behavior of the mechanical system [29,30]. In contrast, steel springs have linear stiffness within a wide range of deformation. However, specific dynamical effects can appear due to the nonlinear relation between the side bending displacement of steel springs and vertical stiffness [31,32]. Coupled mode parametric resonance in a vibrating screen is investigated on the two mass models in [33]. The additional disturbances in the measured signals and dynamical effects can be produced by the inertial vibrators’ bearings [34], damage and wear from which can result in a complex vibration spectra structure.

The most desirable by the energy consumption are resonance vibrating screens [35] but they are not widely used in the industry due to working mode instability and complexity of control when material mass is changing. Issues of multiple vibrators’ synchronization are considered in [36] and many other works. A chaotic vibrating screen is proposed in [37] to improve its sieving performance.

In the standard far beyond resonance vibrating screens, a serious dynamical problem causing excessive energy consumption constitutes the Sommerfeld effect when the machine structure passes its main resonance at startup. About 30% of the additional power of electric drives is needed for reliable transition through the resonance, which results in excessive energy consumption at the working rotation speed. When the rotation speed of vibrators goes down, the screen structure passes resonance very slowly and its elements are subjected to excessive loading cycles of high amplitude. Some solutions are proposed in this regard based on magneto-rheological dampers (MRDs) [38,39] and electric drive control [40], which is not yet widely implemented in the industry.

The additional dynamical loading is caused by the gaps opening, which can appear due to contact wear in joints and deformation of bolts. This effect has a potential threat to appear in non-stationary vibrating machines with reverse loading. Thus, due to the significant influence on the lifetime and reliability of different parts of the vibrating screen, clearances may be thought of as the most critical but hidden for measurement operational parameters. The bolted joints are one of the most vital factors determining the correct operation of vibrating screens. The strength capability of bolted joints supposes that the designers of machines should forecast their peak loads and prevent the contacted surfaces from opening at any joint under any forces because in this case, the additional stresses (torsional, bending, shear) appear in the bolt shank [41]. The greater preloading (limited by yield stress) can reduce the chance of bolt failure. Although preloading of bolted joints is the crucial factor, the specific tightening torque is not usually available during the repairing process, especially for large-size industrial systems where bolts diameter can reach 100 mm. Based on the ASTM F568M, the metric bolts used for heavy-duty applications should have property classes 8.8, 10.9, or 12.9 and be made of alloy steels. However, in practice, the strength capacity of bolts can significantly deviate from the standard values due to different factors.

### 1.2. Diagnostics of Bolted Joints Loosening in Structures

The diagnosis of bolts loosening is a complex scientific and engineering problem considering the potentially massive number of bolted joints and the dramatic outcomes of their loosening, leading to the unexpected redistribution of inner loads between other bolts in joints, not only in rotating systems but in building, bridge, and other infrastructure that use a lot of bolts. Health monitoring of bolts can be divided into two general approaches. The first is based on smart bolts and dense sensors in the local area, and the second approach uses analysis of bolts based on modal analysis of whole structures and systems. A dense sensor approach can be performed by measuring tensile stress with ultrasonic waves [42,43], utilizing acoustic emission on rotating systems [44], electrical conductivity [45], vibroacoustic modulation (VM), and wave energy dissipation (WED) [46,47,48], as well as using spectral sidebands and high-order harmonics [49]. For measuring, such instrumentation can be used as piezoelectric active sensing [50,51,52,53], smart washer manufactured by lead zirconate titanate (PZT) [54,55,56,57]. Also, these methods can be combined with wireless technology for critical civil infrastructure and industrial facility monitoring. In the sieving screens, strong continuous excitation from unbalanced vibrators may have an influence on the reliability of electronic components.

In recent years, due to technological improvement of cameras and related algorithms, the approach based on image processing to diagnose bolts has accumulated some favor [58,59,60]. This method is usually combined with deep learning technology [61,62,63], support vector machines [64], and nonlinear decomposition such as empirical mode decomposition [65]. Selecting the reference points to detect changes in the nuts’ position has a substantial effect on the reliability of these methods. Nevertheless, these methods cannot detect phenomena such as bolt creep and axial deformation in some states.

Another category of bolt diagnostic approaches is based on identified modal parameters of the system. Detecting changes in a natural frequency and phases can detect the weak bolt tightening caused by changing the system’s stiffness and contact friction [66]. For instance, the vibration transmissibility function is a more reliable parameter to determine the joint state, while natural frequency and modal damping in modes with low frequency seemed less reliable. In other studies [67,68], experimental and theoretical methods have been accomplished to assess system modal properties and frequency response functions to detect bolted joint degradation [69,70,71]. In [72], the authors demonstrated that the first-order phase difference parameter is susceptible to the looseness of the bolts on the plate joint of the wind turbine tower. In [73], a finite element simulation is used to analyze rod flange-bolt structure unit (FBSU). Authors in [74] developed a simple method to assist bolt tightness by measuring natural frequency and damping ratio during the hammer test. In [75], non-linearity and damping ratio in bolt frequency response were studied. In [76], the authors modelled the bolt as a plane beam with two linear end springs (transverse and rotational).

In this research, the authors are focused on the dynamical analysis of a vibrating screen with an emphasis on the causes of frequent failures on the heavy upper deck and methods of bolted joint diagnostics. All further calculations are regarding only those bolts that fasten the upper deck to the screen structure to restrain its vertical displacement but not the other bolts in side panels, which loosening is planned for analysis in our future research. We consider the most difficult case when nuts are fixed and other methods, e.g., visual inspection, are not sufficient in the bolted joint maintenance on the screens.

Both practical and theoretical goals motivated this research. The practical goal was to understand the reasons for frequent failures of bolted joints in the vibrating screen and to develop remedies against such failures. The theoretical goal was to develop methods of bolted joint loosening based on a dynamical model and implement the appropriate signal processing techniques. In fact, the developed method and diagnostic parameters are applicable to any horizontal screens and other vibrating machines (feeders, transportation tables) where two main parts are joined by the bolts and susceptible to frequent failures.

The scope of the conducted research covers industrial research and measurements on the fully functional laboratory screen. The novelty of the developed approach is in considering the vibrating screen as a system with a changeable structure when an additional degree of freedom appears due to bolted joint degradation. In this case, the second mode natural frequency change and the first mode damping values are used as the diagnostic parameters of bolted joint loosening. In addition, anti-resonances are identified in the 2DOF dynamical system and their possible effect on screen working efficiency and diagnostics is explained. The developed method is not sensitive to the non-Gaussian noise created by the sieved bulk material.

The paper is organized as follows. In Section 1, we represented all aspects of vibrating screens’ operation and their diagnostics. In Section 2, we give a description of the investigated machines. In Section 3, the dynamical model, modal analysis, and detuning from resonances are represented. In Section 4, the results of model simulations are given, including non-Gaussian noise. In Section 5, the results of measurements on a laboratory screen are represented to validate the new diagnostic method. Finally, in Section 6, the achieved results are discussed, and in Section 7, conclusions and further research are highlighted.

## 2. Materials and Methods

**Figure 1:**
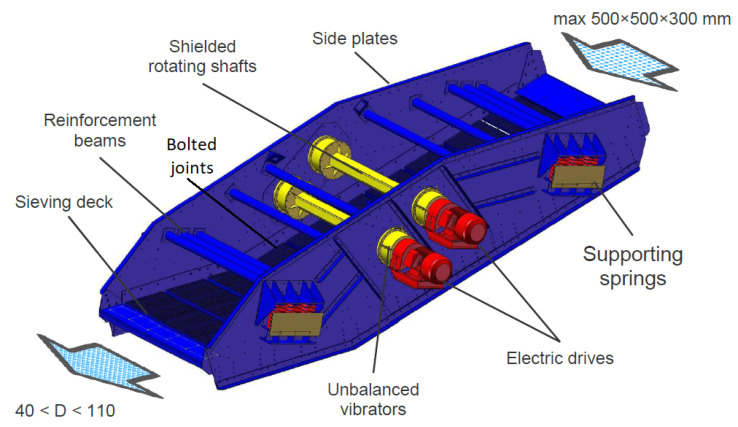
The typical design of a vibrating sieving screen with two unbalanced vibrators [77].

**Figure 2:**
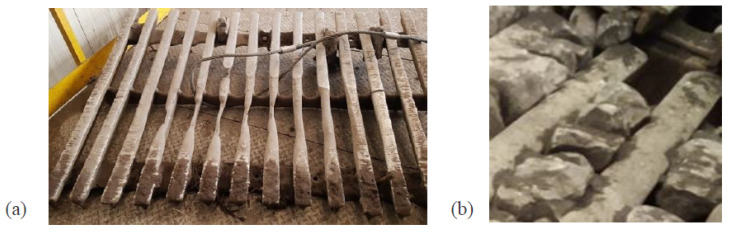
Upper deck of vibrating screen: severe abrasive wear of beams (**a**); blinding with a near-mesh-size material (**b**).

In our research, we analyze two devices. The first is an industrial vibrating sieving screen, a general view of which is depicted in Figure 1. The second machine is a fully functional sieving screen in our laboratory, a photo of which is shown in Section 5. Maintenance records and some vibration data from the industrial screen are observed for a long time to undermine its weak elements (see statistics in Figure 2). In particular, the bolted joints are determined (26% of failures), which motivated our studies on their diagnostics. In addition, the spring–mass parameters of this screen (see in Section 2.2) are used in the dynamical model simulations. The second machine was a laboratory screen, which was used to validate our method of diagnostics since we could not conduct experiments with controlled bolt loosening and measurements in the industrial plant.

### 2.1. Design of Industrial Sieving Screen

The investigated typical industrial vibrating screen (see Figure 1) includes the body with side panels linked by reinforcement beams, one or multiple sieving decks, and helical supporting springs. All screen parts are subjected to significant abrasive wear and fatigue; thus, instead of welded joints, special huck bolts are used.

### 2.2. Vibrating Screen Dynamics and Failures

The upper deck blinding with a near-mesh-size material can increase the weight of vibrating masses while the severe abrasive wear of beams will reduce the mass of the deck (see Figure 2). The total mass of the deck obviously includes a certain mass of sieved material (up to 30% of total mass) at every moment in time. Nevertheless, vibrating screens can be considered systems with constant lumped stiffness and mass parameters at a certain period of their operation. Instead, the bolted joints—the subject of research—can change axial stiffness in the course of machine operation, but they are also assumed as constant parameters at a certain moment in time.

**Figure 3:**
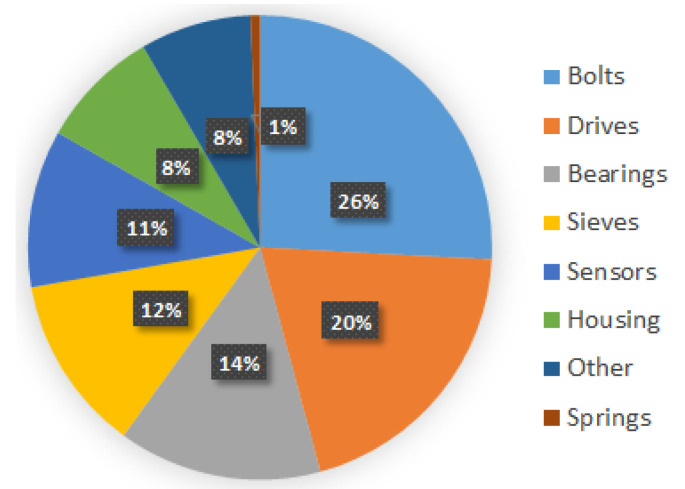
Statistics of elements failures in vibrating screen.

According to the recommendations of the vibrating screens’ producers regarding the equipment maintenance actions (see Table 1), the vibrator bearings’ and sieves’ mounting joints are the most frequently inspected units, which means they have the highest susceptibility to failures and malfunctions. This is justified by the repair data analysis (see Figure 3) where bolts, drives, bearings, and sieves constitute 72% of all issues that happen during the vibrating screen operation. Hence, the diagnostics of these elements is of great importance for the smooth operation of industrial enterprises.

### 2.3. Measuring Equipment

Vibration measurements were obtained by the Kistler LabAmp 5165A, a 4-channel universal laboratory charge amplifier used for dynamic signal or mechanical quantity measurements with piezoelectric sensors (IEPE) [78]. The K-Shear 8702B500 accelerometers coupled with the amplifier were used. The parameters of the sensors were as follows: sensitivity—10 mV/g; maximum acceleration range—500 g.

## 3. Methodology of the Bolted Joints Diagnostics in Vibrating Screens

### 3.1. Equation of Motion

The equation of the mechanical system motion with *n* degrees of freedom and viscous damping can be represented as follows:

(1) $$\left[m\right]\ddot{x}+\left[b\right]\dot{x}+\left[k\right]x=F\left(t\right)$$

where, *x*, $\dot{x}$, and $\ddot{x}$ are displacment, velocity, and acceleration, respectively. In addition, [m], [b], and [k] are mass, damping, and stiffness matrix, respectively, and *F* is force. Also, the mentioned equation can be presented in a state space form [79]:

(2) $$\dot{x}\left(t\right)=\dot{x}\left(t\right)$$

(3) $$\ddot{x}\left(t\right)=-{\left[m\right]}^{-1}\left[b\right]\dot{x}\left(t\right)-{\left[m\right]}^{-1}\left[k\right]x\left(t\right)+{\left[m\right]}^{-1}F\left(t\right)$$

by definition of 2n-dimensional state space vector y(t) as follows:

(4) $$y\left(t\right)=\left\{\begin{array}{c} x(t)\\ \dot{x}\left(t\right) \end{array}\right\}$$

Equations (2) and (3) can be rewritten in a state space form as follows:

(5) $$\dot{y}\left(t\right)=\left[A\right]y\left(t\right)+\left[B\right]F\left(t\right)$$

where the coefficients matrices [A] and [B] of order 2n×2n and 2n×n, respectively, are equal to:

(6) $$\left[A\right]=\left[\begin{array}{cc} 0 & I\\ -{\left[m\right]}^{-1}\left[k\right] & -{\left[m\right]}^{-1}\left[b\right] \end{array}\right]$$

(7) $$\left[B\right]=\left[\begin{array}{c} \left[0\right]\\ {\left[m\right]}^{-1} \end{array}\right]$$

#### 3.1.1. Modal Analysis

For the modal analysis, first, we assume the free vibration problem with f(t)=0, so Equation (5) can be rewritten as follows:

(8) $$\dot{y}\left(t\right)=\left[A\right]y\left(t\right)$$

This equation represents a set of 2n first-order ordinary differential equations. The solution Equation (8) is considered in the following form:

(9) $$y\left(t\right)=Ye^{\lambda t}$$

where *Y* and λ are constant vectors and values, respectively. By substituting Equation (9) to Equation (8) and simplifying it, we obtain

(10) [A]Y=λY

Equation (10) is a standard algebraic eigenvalue problem. The solution of it gives the eigenvalues and corresponding eigenvectors. Following the previous studies, the natural frequencies of the two lowest modes of vibrations in heavy industrial machines are rarely observed above 20–30 Hz and are sensitive to clearances in the dynamical system.

#### 3.1.2. Damping Ratio

Besides the natural frequencies, the damping ratio is used for bolt looseness detection. The method is based on the transient vibration curve robust nonlinear fitting solved with the least-square approach. The problem of finding coefficients *x* can be defined as follows:

(11) $$\min_{x}{∥F\left(x,xdata\right)-ydata∥}_{2}^{2}=\min_{x}\sum_{i}{\left(F\left(x,xdata_{i}\right)-ydata_{i}\right)}^{2}$$

where *xdata* is input data, *ydata* is an observed output, both represented as either matrix or vector, and F(x,xdata) is a matrix-valued or vector-valued function of the same size as *ydata*, which is computed instead of a standard sum of squares. Using this method of curve fitting, a damping ratio is estimated by the decaying transient vibrations to detect bolt loosening during experiments.

#### 3.1.3. Frequency Response Functions

Modal testing is an experimental technique that is used to design the modal model of linear invariant vibratory systems over time. The theoretical basis of the technique is to determine the relationship between one location’s vibration response and another location’s excitation as a function of the excitation frequency. This relationship is often a complex mathematical function, called the frequency response function (FRF) [80]. FRFs are often displayed in acceleration units (g) and force units (N), resulting in g/N units. Also, there are other formats in which FRF can be displayed. These alternative formats are created by executing mathematical operations over the FRF—integration or differentiation, then, acceleration can be replaced with velocity and displacement.

In the following, we show the FRF representation by using displacement based on Equation (1), which is known as compliance:

(12) FRF(ω)=x/F

where *x*, *F*, and ω are displacement, force, and frequency, respectively. More information about the driving procedure and details can be found in reference [80].

### 3.2. Impulsive Non-Gaussian Noise

It is known from the theory and authors’ experience of industrial measurements that stochastic non-Gaussian impacts from the large pieces of sieved material falling on the upper deck produce a wide-band spectrum of forces. It can be described by the following formulas [81]:

(13) $$X=S_{\alpha ,\beta }\times \frac{\sin\left(\alpha \left(V+B_{\alpha ,\beta }\right)\right)}{{\left(\cos V\right)}^{1/\alpha }}\times {\left(\frac{\cos\left(V-\alpha \left(V+B_{\alpha ,\beta }\right)\right)}{W}\right)}^{(1-\alpha )/\alpha }+\mu$$

(14) $$S_{\alpha ,\beta }=\sigma \times {\left[1+{\left(\beta \tan\frac{\pi \alpha }{2}\right)}^{2}\right]}^{1/2\alpha }$$

(15) $$B_{\alpha ,\beta }=\frac{\arctan\left(\beta \tan\frac{\pi \alpha }{2}\right)}{\alpha }$$

(16) $$X=\sigma \times \frac{2}{\pi }\left[\left(\frac{\pi }{2}+\beta V\right)\tan V-\beta \log\left(\frac{\frac{\pi }{2}W\cos V}{\frac{\pi }{2}+\beta V}\right)\right]+\frac{2}{\pi }\beta \sigma \log\sigma +\mu$$

### 3.3. Dynamical Model of the Vibrating Screen

**Figure 4:**
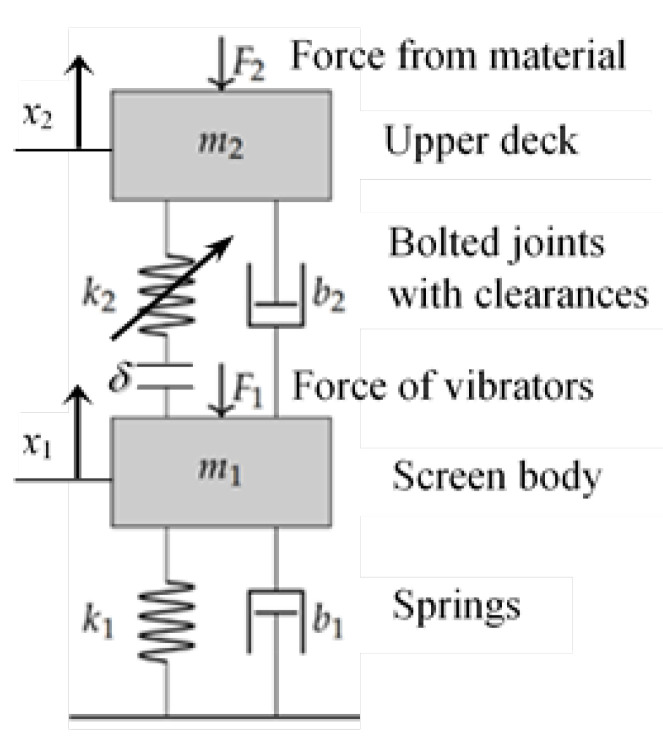
The calculation scheme of the large-scale industrial vibrating screen with the upper sieving deck as a separate mass (see parameters’ description in Table 2).

For vibrating screen analysis, the 2-DOF dynamical model is assumed, the calculation scheme for which is represented in Figure 4. The separation of the second mass corresponding to the upper deck is quite justified because its size and weight (about 5 t) are of the same order as the rest of the screen body. This is a distinctive feature of the developed dynamical model, since the screen is usually considered a single mass.

The matrices of the dynamical system parameters in Equation (1) have the following form:

(17) $$\left[m\right]=\left[\begin{array}{cc} m_{1} & 0\\ 0 & m_{2} \end{array}\right],\left[b\right]=\left[\begin{array}{cc} b_{1}+b_{2} & -b_{2}\\ -b_{2} & b_{2} \end{array}\right],\left[k\right]=\left[\begin{array}{cc} k_{1}+k_{2} & -k_{2}\\ -k_{2} & k_{2} \end{array}\right]$$

which values are given in Table 2.

The governing equations for the 2-DOF non-linear system are the same as for the linear model but the stiffness of degrading bolts is described by the discontinuous nonlinear function with a dead zone (gap) and two linear pieces of constant stiffness:

(18) $$k_{2}=\left\{\begin{array}{c} 0,\\ k_{2},\\ k_{3}, \end{array}\begin{array}{c} \delta <\Delta_{1}\\ \Delta_{1}\leq \delta \leq \Delta_{2}\\ \delta >\Delta_{2} \end{array}\right.$$

where $\delta =(x_{2}-x_{1})$ is the amplitude of bolts’ tensile deformation; $\Delta_{1}$ is the deformation corresponding to the yield stress of bolts’ material; $\Delta_{2}$ is the deformation corresponding to the gap opening in the bolted joints. Material hardening is not assumed for stresses above the elastic limit for high-strength steel bolts.

The composed system of non-linear differential equations is solved numerically by the Runge–Kutta method of fourth order with a constant time step of integration.

This model is further used for vibrating screen dynamics analysis and diagnostic feature investigation.

## 4. Results of Simulation

### 4.1. Analysis of Frequency Response Functions

**Figure 5:**
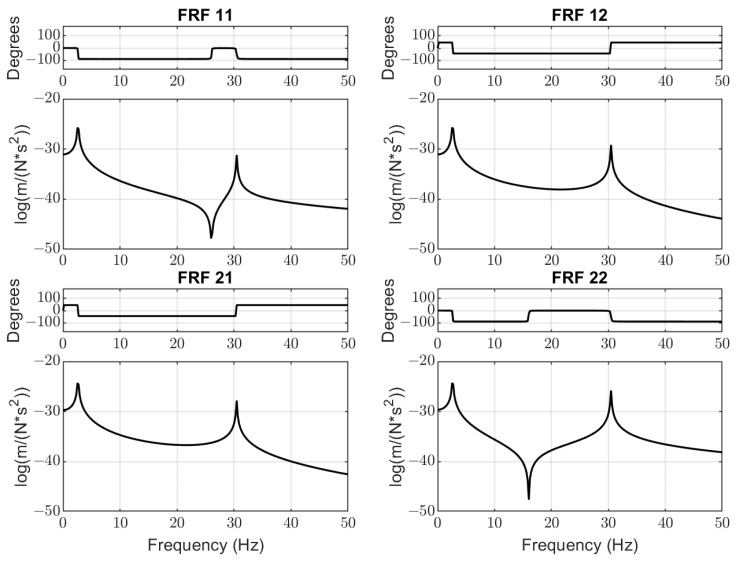
Frequency Response Functions (FRFs) of the vibrating screen by the four channels.

The dynamics analysis included the building of Frequency Response Functions (FRFs) of the vibrating screen. Taking into account that the total exciting force *F*_1_ from both unbalanced vibrators acts directly on mass *m*_1_ and impacts force *F*_2_ from the falling pieces of the material acting on mass *m*_2_, the following four channels are considered (see Figure 5):

- *FRF*${}_{11}$: from (vibrators’ force *F*_1_) to (displacement of mass *m*_1_);
- *FRF*${}_{12}$: from (vibrators’ force *F*_1_) to (displacement of mass *m*_2_);
- *FRF*${}_{21}$: from (material impacts force *F*_2_) to (displacement of mass *m*_1_);
- *FRF*${}_{22}$: from (material impacts force *F*_2_) to (displacement of mass *m*_2_).

The exact values of natural mode frequencies of vertical vibration are as follows: 1st mode—2.6 Hz; 2nd mode—30.5 Hz. Functions *FRF*${}_{11}$ and *FRF*${}_{22}$ have anti-resonance frequencies of about 26.2 Hz and 16.1 Hz, respectively. This means that the working frequency of vibrators should be at least ±5 Hz from the minimum point of *FRF*${}_{11}$ to avoid the inefficient energy consumption by the electrical motors of vibrators.

**Figure 6:**
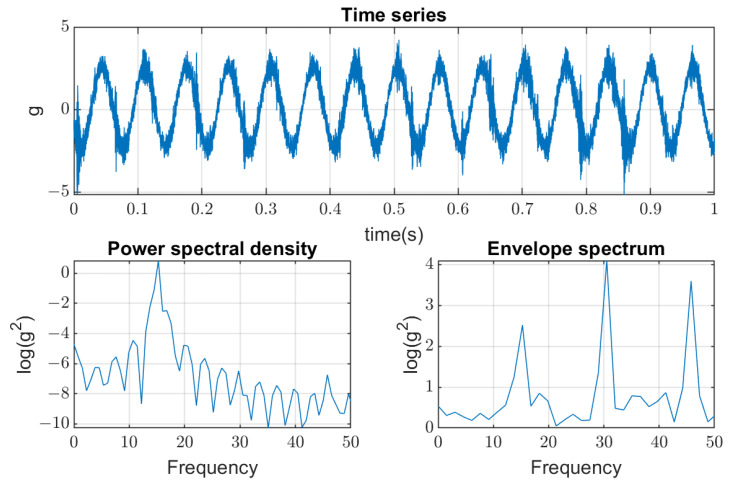
Spectrum with higher harmonics of vibration measured on the shaft bearings of unbalanced vibrators.

While the first mode of vibration is mainly determined by the design parameters of the screen (total mass and stiffness of springs in the supporting units), the second mode of the screen’s natural vibration depends on several factors. The most influencing factor is the gradually changing stiffness between mass *m*${}_{1}$ and mass *m*${}_{2}$, which greatly depends on bolted joints’ condition (tightening and axial plastic deformations). The mass of sieved material on the screen decks has less influence on the second vibration mode. It was noted by the data obtained from the permanent vibration monitoring system that the spectrum of excitation force measured on the bearings of the vibrators’ shafts contains higher harmonics (30 Hz, 45 Hz) at certain periods of screen operation. This feature corresponds to the bad condition of one or several bearings or supporting springs (see Figure 6).

After maintenance actions are undertaken on the screen, those higher harmonics disappeared, which proves their origin. Measurements of vibration in the four corners of the main screen housing on supporting springs showed that it vibrates as the rigid body. Hence, even one bearing with damaged rings or having excessive clearance can generate not only 15 Hz but as high as 30 Hz and 45 Hz harmonics and excite the second natural mode with increased tension in the bolted joints. Since the maintenance actions of bearings and bolted joints have different periods, this process can occur at any time of operation and requires new methods of damage detection in the condition monitoring system.

### 4.2. Dynamical Model Simulation

**Figure 7:**
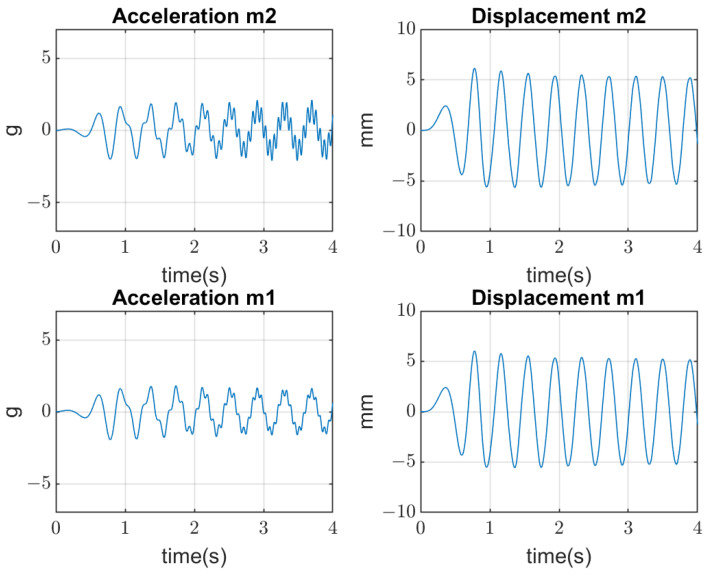
Simulation of 2-DOF screen vibrations and bolted joints with tightened bolts in good condition.

**Figure 8:**
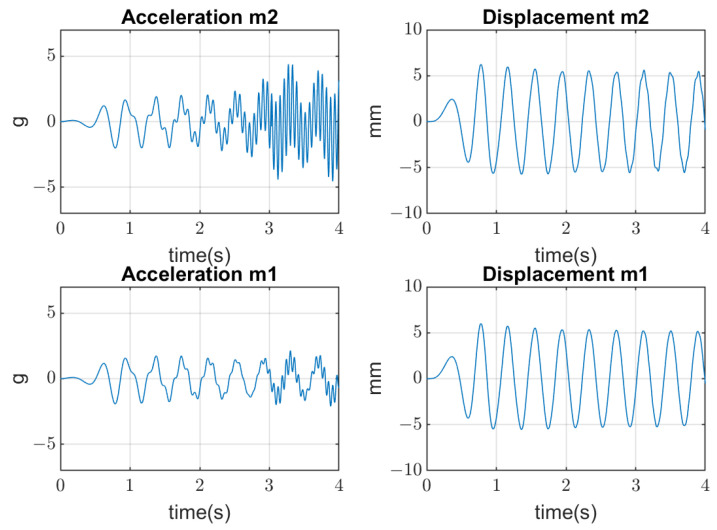
Simulation of 2-DOF screen vibrations and bolted joints with bolts in moderate condition.

**Figure 9:**
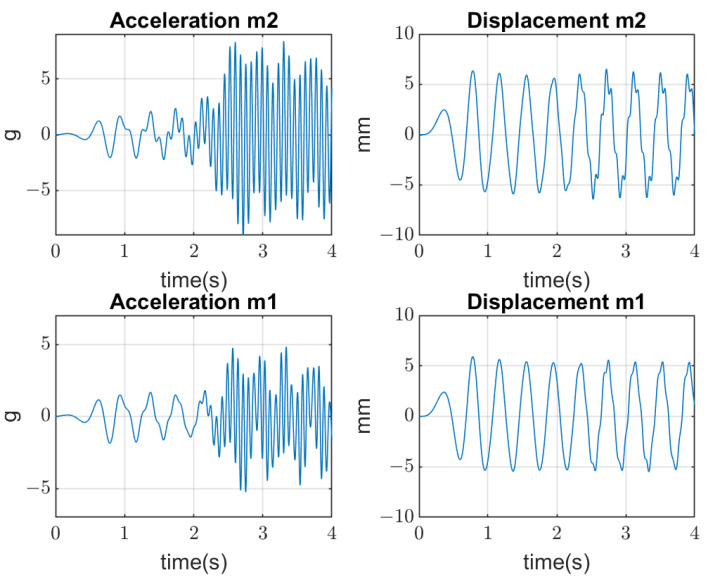
Simulation of 2-DOF screen vibrations and bolted joints with contact opening at high amplitudes of forces.

The vibrating screen simulations are conducted in several aspects. Firstly, the periodical excitation is applied to mass $m_{1}$ by the real forces acting in the industrial screen. Three cases are considered: (1) when bolts are tightened (linear stiffness $k_{2}$); (2) when the amplitude of vertical forces is beyond the yield stress of the bolts’ material; and (3) when the clearances appear with joints’ opening, which produces an additional impact on the bolted joints (non-linear stiffness $k_{2}$) and their further quick degradation until breaking. Results of simulations are represented in Figure 7, Figure 8 and Figure 9, where amplitudes of both masses in the time domain are represented. The values of mass $m_{1}$ displacement are verified by the real data of measurement (about 5–8 mm).

**Figure 10:**
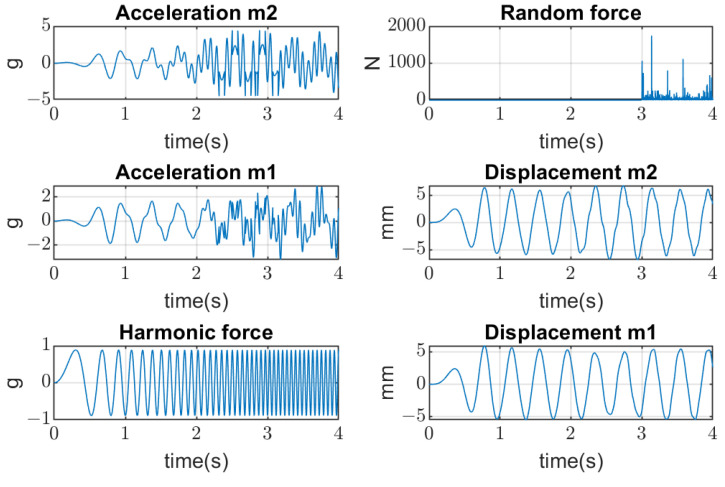
Transient processes of screen vibrations resulted from stochastic non-Gaussian impacts on the upper deck.

In the second stage of simulations, the stochastic impacts are applied to the mass $m_{2}$ generated by the falling pieces of material, which have non-Gaussian distribution. The transient processes of the mass $m_{2}$ vibration and mass $m_{1}$ are represented in Figure 10.

**Figure 11:**
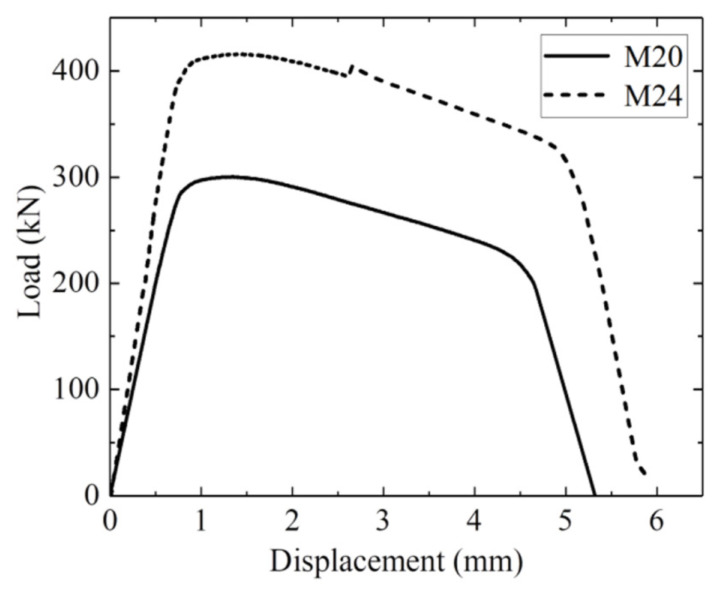
The diagram of tensile stress relation with bolted joint deformation [82].

**Figure 12:**
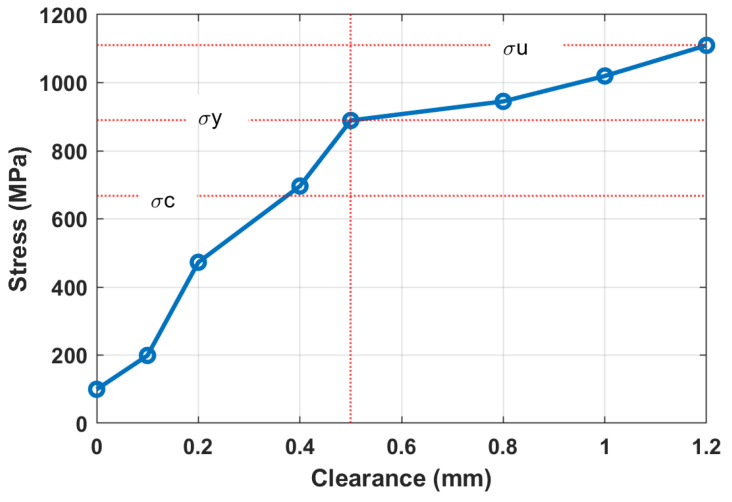
Tensile stress relation with bolted joint loosening (gap size) between the upper deck and the vibrating screen body (the vertical red dotted line shows a maximum allowable clearance of 0.5 mm, which corresponds to achievement of yield stress in the bolts).

The mechanism of bolted joints loosening can be explained as developed in several steps and influenced by many factors. The initial tightening force (torque) is recommended to provide uniform clamping stress $\sigma {}_{C}$ about 75% of the yield stress $\sigma {}_{Y}$. During the first period after repair, bolts work within the linear range of elastic deformation, which is defined by the difference of separate masses’ displacements ($x_{2}-x_{1}$). See the high-strength bolt testing diagram in Figure 11 from the work [82]. During the period of gradual deterioration, bolts are subjected to periodic forces from the vibrators, but the upper deck and the whole screen body still move in phase. At the next stage of degradation, due to additional impacts from big pieces of the treated material falling on the upper deck, the amplitude of bolts’ tensile forces reaches the yield stress $\sigma {}_{Y}$ of the bolts’ material, and plastic deformation appears, which creates gaps in the joints. With increasing clearances, the upper deck and screen body start to move out of phase, the ultimate stress $\sigma {}_{U}$ is reached in certain bolts, and failures occur. The results of dynamical model simulations are described in Figure 12 where the tensile stress of bolts is given in relation to different clearance sizes.

The critical value of clearance is about 0.5 mm, after which the failure is unavoidable. In practice, with two accelerometers installed on the screen body and on the sieving deck, tensile forces (stress) can be easily calculated with relative displacements (deformation), which are then applied to the diagrams in Figure 11 and Figure 12. Such interpretation and visualization of the bolts’ degradation process help the maintenance staff to undertake repair actions in time to prevent an abrupt failure and unplanned machine downtime.

**Figure 13:**
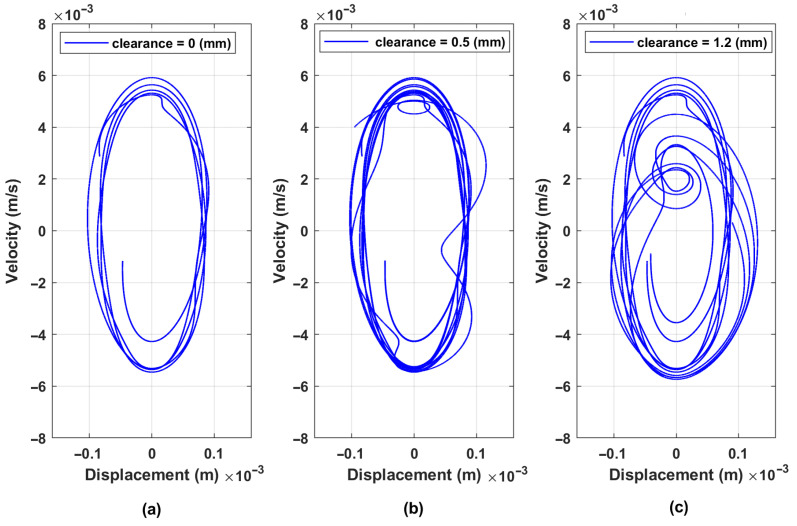
Phase space plots of screen upper sieve (mass $m_{2}$) vibration for three cases of clearances: (**a**) normal state (0 mm); (**b**) moderated bolt looseness (0.5 mm); and (**c**) critical bolt looseness (1.2 mm).

In addition, for bolt looseness detection, the phase space plots (PSPs) are built in Figure 13. Three cases are shown there—normal state (0 mm), moderated bolt looseness (0.5 mm), and critical bolt looseness (1.2 mm). The explicit changes in PSP are observable in the graphs, which are caused by the non-linear characteristics of the bolted joints’ stiffness when clearance appears.

**Figure 14:**
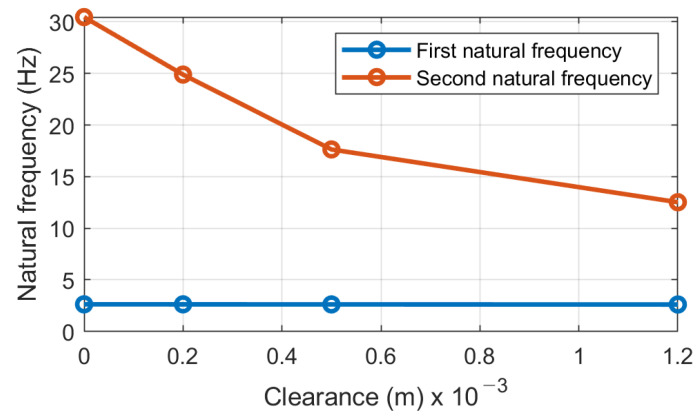
The dependence of the second natural mode frequency on the clearance in the bolted joints.

Based on the results of simulations, we can conclude that the modal parameters of the system should have high sensitivity to clearance in the bolted joints between two masses of the screen—the main body and the upper sieving deck. The dependencies of the first and second natural modes’ frequencies on the clearance in the bolted joints are shown in Figure 14. In the range of small values of clearances (up to 0.5 mm), sensitivity is estimated at about 25.6 Hz/mm. The sensitivity is decreasing for the larger values of clearances but remains high enough (about 7.3 Hz/mm) for practical application. Since the excitation force is determined by the pre-installed eccentricity of the constant unbalanced masses of vibrators, the sensitivity will not be affected for a certain screen design and settings.

Besides the natural frequencies, damping ratio and PSP were taken as the diagnostic parameters.

### 4.3. System Detuning from the Resonance

When the residual plastic deformation increases enough, clearances appear in the joints, and the amplitude of the second natural mode increases, which corresponds to the out-of-phase motion of the upper deck and the screen body. In the case of simultaneous defects or excessive clearances in the bearings of vibrators, the higher harmonics of excitation force appear in the spectrum, which greatly increases amplitudes of the second mode, and hence tensile stress in the bolts. Finally, loosened bolted joints induce contact opening in the upper deck around the places of its mounting on the screen housing. The worst stage of degradation starts when an initial crack appears due to the unequal loads on separate bolts above the ultimate stress of their steel grade.

Moreover, the upper deck of the screen consists of three sections with similar masses. Every section has different angles of inclination and different amplitudes of the vertical components of forces. Therefore, the above-mentioned stages of worsening bolt condition will develop asynchronously, requiring even more frequent maintenance actions. The bolts loosened in one section will have an impact on the other two sections. Hence, scheduled maintenance is better when conducted for the whole set of bolts on the upper deck. All issues mentioned above require the system to detune from the resonance at the second natural node frequency.

**Figure 15:**
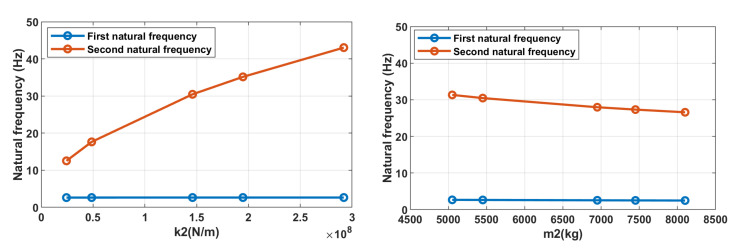
Dependence of the natural modes’ frequencies on the stiffness $k_{2}$ of bolted joints and mass $m_{2}$ of upper deck.

The dependencies of the first and second mode natural frequencies on the $k_{2}$ stiffness and mass of upper deck $m_{2}$ are given in Figure 15. The sensitivity of the second natural mode frequency to stiffness $k_{2}$ changes is about $1.14\times 10^{-7}$ Hz/(N/m), and to $m_{2}$ it is about $-1.60\times 10^{-4}$ Hz/kg. The first natural mode frequency is almost insensitive to those parameter changes. Hence, to tune out the vibrating screen from the resonance by the second natural mode around the 2×, 3× harmonics (30, 45 Hz) of the main excitation frequency (15 Hz), it is required to change the total stiffness of bolted joints by $2.63\times 10^{7}$ N/m, which corresponds to the frequency deviation by at least ±3 Hz.

Since the design diameters of the bolts provide the required strength capacity, their decrease is not possible, nor is the section’s increase (restricted by hole diameters). Instead, a decrease in the bolted joints’ stiffness and simultaneously providing better damping of shock impacts in joints of the upper deck can be achieved by the special pads made of polyamide or other durable elastic material installed under bolts’ heads and nuts. The geometry of such pads should be calculated based on applied material properties and design.

**Figure 16:**
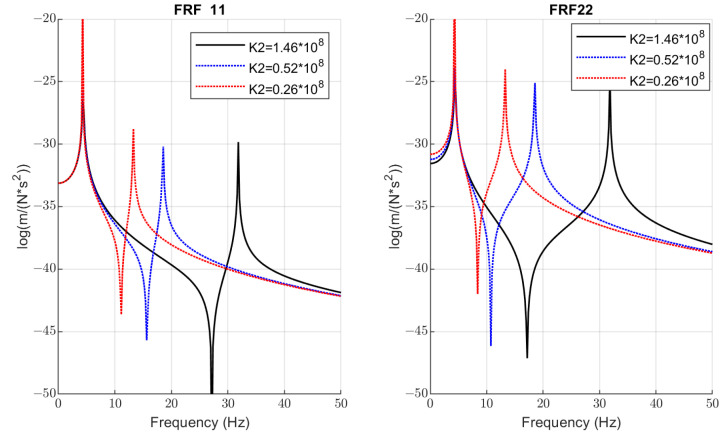
The dependence of *FRF*${}_{11}$ and *FRF*${}_{22}$ on the bolts’ stiffness $k_{2}$ (grades of loosening).

The relations of *FRF*${}_{11}$ and *FRF*${}_{22}$ with the bolts’ stiffness $k_{2}$ (grades of looseness) are represented in Figure 16. The anti-resonances observed on these graphs are the distinctive features of a 2-DOF dynamical system. Their frequencies do not coincide with the vibrator rotations in the investigated screens; however, they allow a better understanding of how to avoid energy leaks. The greater stiffness (better tightening of bolts and their condition) corresponds to a deeper drop in amplitudes at these frequencies and greater energy leaks may occur. For the intermediate value of stiffness $k_{2}=0.52\times 10^{8}$ (blue line on the *FRF*${}_{11}$ graph in Figure 16), the anti-resonance frequency (15.62 Hz) is very close to the vibrators’ rotation frequency (15 Hz).

In the context of bolted joints’ diagnostics, the coincidence of the vibrators’ rotation and anti-resonance frequency has a positive effect on the dynamics reduction of bolted joints, because amplitudes of out-of-phase vibrations are less at these frequencies. However, this is not desirable for energy saving of the screen and has to be avoided in practice.

## 5. Measurements on a Laboratory Vibrating Screen

**Figure 17:**
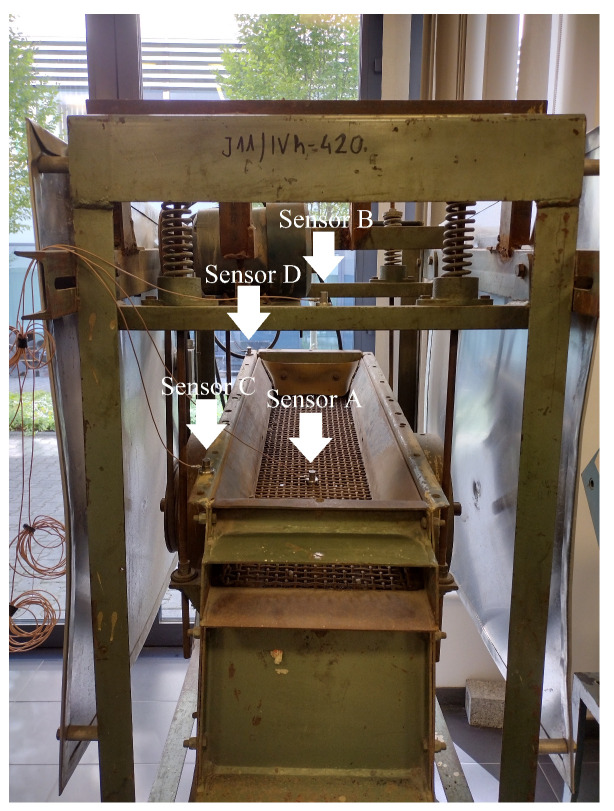
The laboratory vibrating sieving screen with one unbalanced vibrator and bolted joints on the upper sieving deck. The sensors’ positions are shown with arrows.

**Figure 18:**
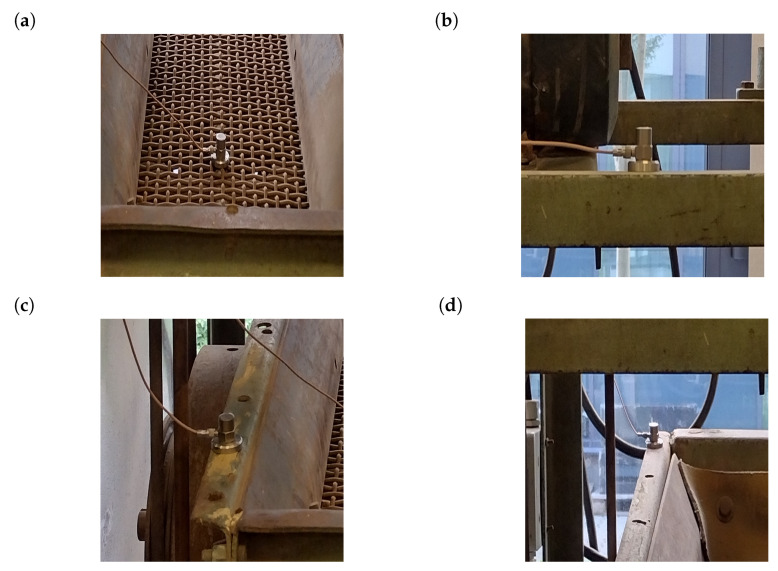
Locations of the mounted sensors: (**a**) sensor A—upper sieving deck; (**b**) sensor B—screen arm; (**c**) sensor C—bottom part of the screen; (**d**) sensor D—upper part of the screen.

Since the measurements of bolt loosening and moreover their regulation is almost impossible in industrial sieving screens, the experimental part of this research was conducted on the fully functional laboratory vibrating screen (see Figure 17) to demonstrate the possibility of bolted joint loosening detection by the vibration signals with the developed methods. Locations of mounted sensors can be seen in Figure 18.

### 5.1. Results of Measurements

**Figure 19:**
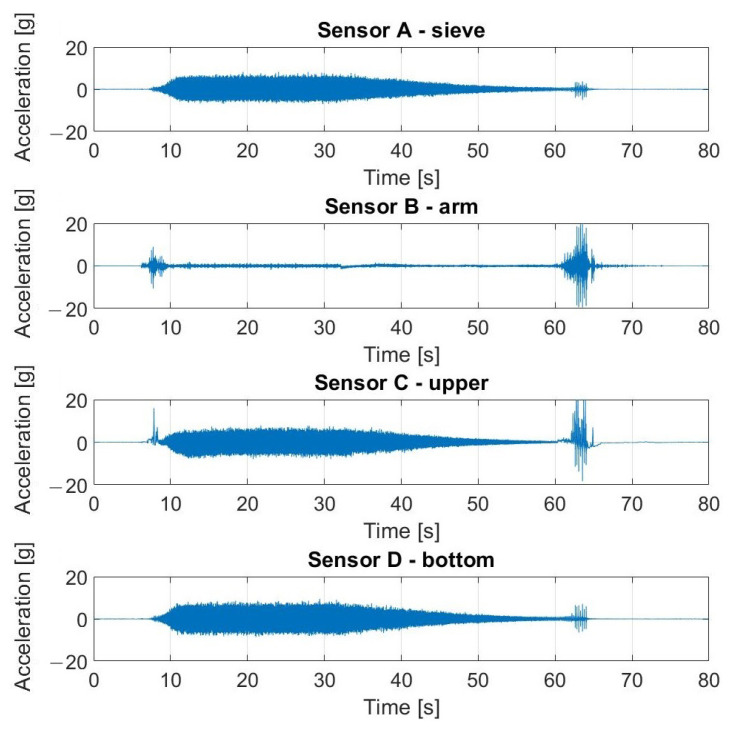
Raw signals from sensors on vibrating screen in normal condition—no loosened bolts.

**Figure 20:**
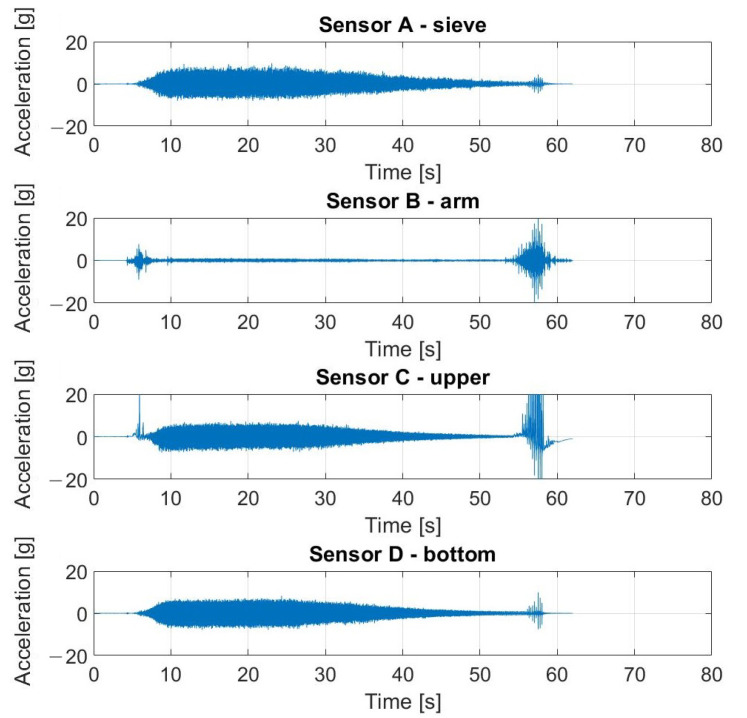
Raw signals from sensors on the vibrating screen, with the left upper screw loosened.

**Figure 21:**
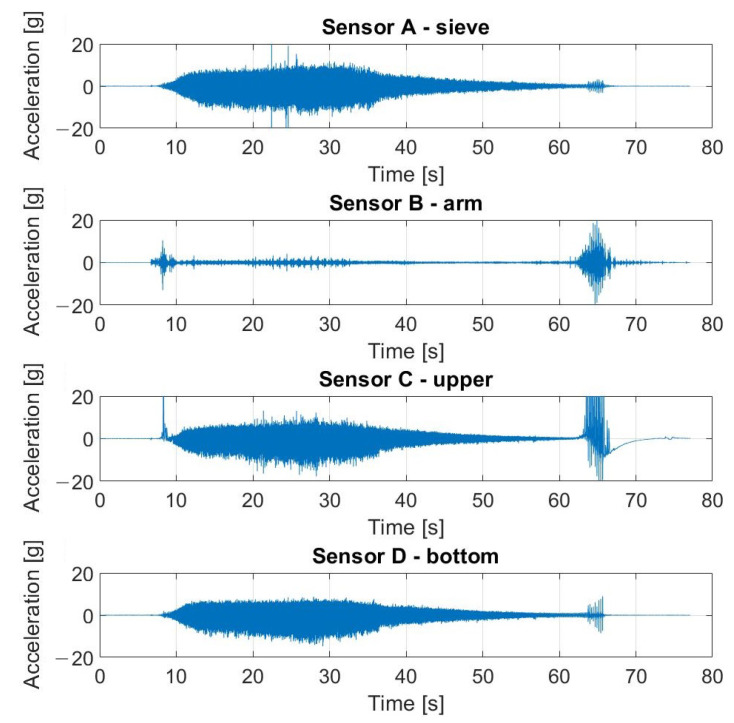
Raw signals from sensors on the vibrating screen, with the left upper screw loosened more than in the previous case.

**Figure 22:**
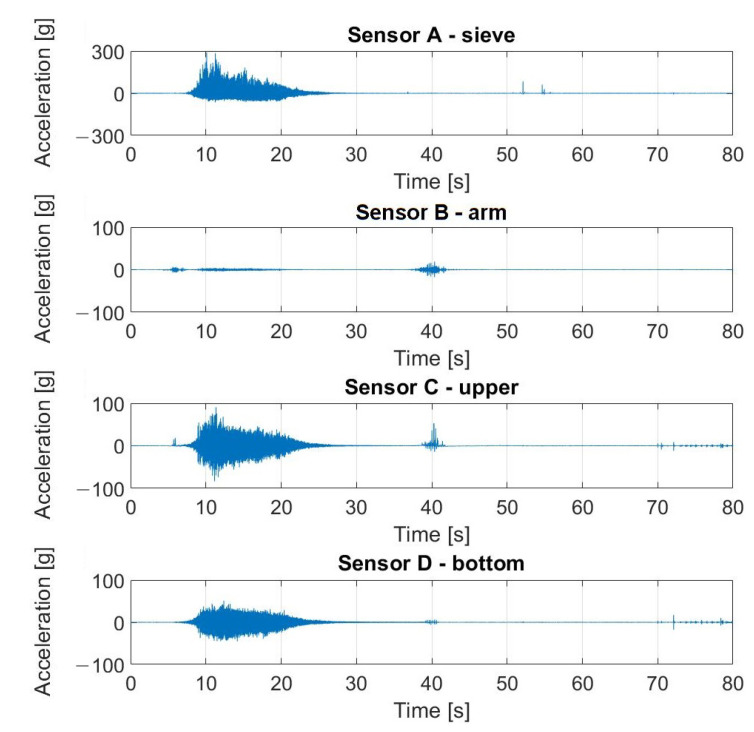
Raw signals from sensors on the vibrating screen with two bottom screws loosened.

The signals registered during the described experiment can be seen in Figure 19, Figure 20, Figure 21 and Figure 22. In the case visible in Figure 22, the measurement was ended early due to the highly increased vibrations and the possibility of damaging the vibrating screen.

**Figure 23:**
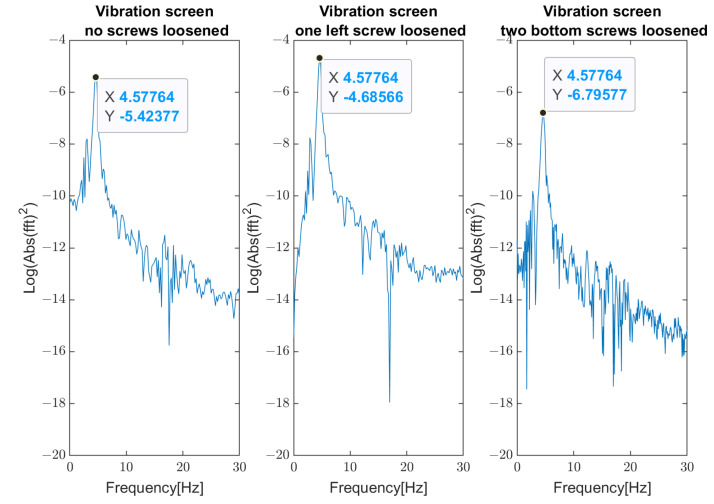
Change of the first natural frequency in three investigated cases (measurements from Sensor A—sieve).

**Figure 24:**
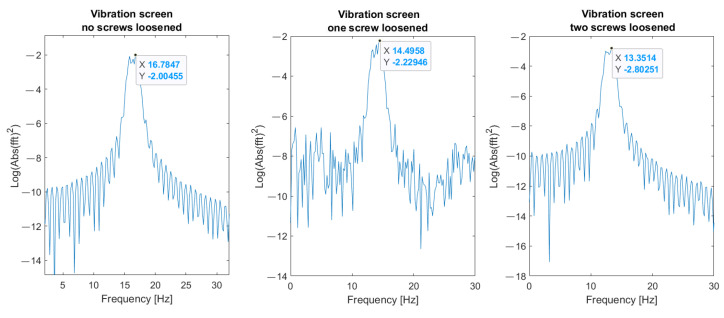
Change of the second natural frequency in three investigated cases (measurements from sensor A—sieve).

Parameters of FFT used in signal processing procedures are as follows: sampling frequency: 25 kHz; data length: 4 s (100,000 samples); frequency resolution: 0.25 Hz; windowing: no window. The spectrum and changes of the first (about 4.2 Hz) and the second (16.7 Hz) natural modes’ frequencies are shown in Figure 23 and Figure 24, respectively. These graphs demonstrate three investigated cases: normal state of bolted joints; one upper left screw is loosened; and two bottom screws are loosened.

**Figure 25:**
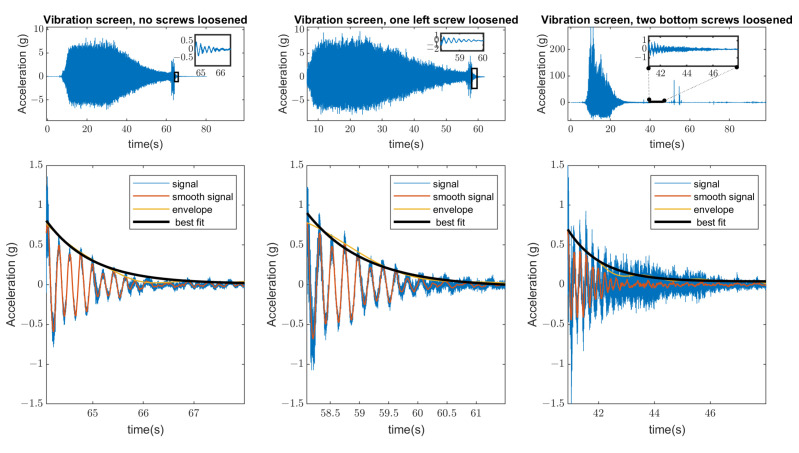
The calculation of the damping ratios in three investigated cases (measurement from Sensor A—sieve) based on exponential approximation of transient signals.

**Figure 26:**
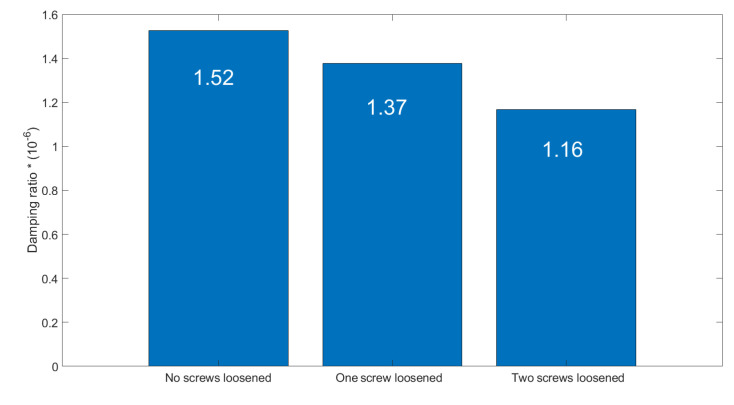
The calculated values of the damping ratio in three investigated cases.

As an additional diagnostic parameter for bolt loosening, the damping ratio was used in the same series of experiments. The change in the damping parameter in three different cases can be seen in Figure 25 and a summary of calculated values is given in Figure 26.

**Figure 27:**
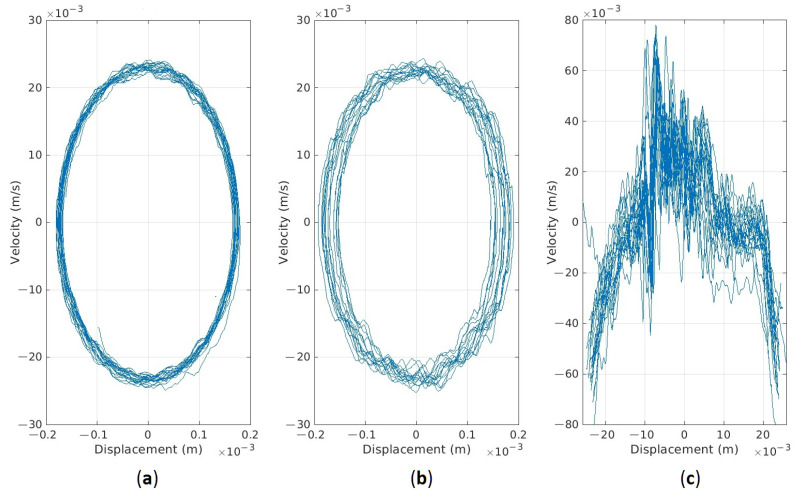
Phase space plots generated on the data from three investigated cases: (**a**) normal state; (**b**) one bolt loosened; (**c**) two bolts loosened (critical state).

As shown above, on the dynamical model of screen vibrations, phase space plots are sensitive to bolt looseness. Based on experimental data obtained on the laboratory screen, these graphs are built for three cases and are shown in Figure 27. In the normal state, PSP is characterized by a trajectory with minimal deviations. The second case with a weak looseness of only one bolt produces visible distortions in the trajectory of the upper sieving deck. The third case demonstrates the critical state (maximal looseness) of two bolted joints when the PSP trajectory is fully degraded and transformed into unpredictable oscillations of high amplitude.

The numerical parameters of PSP shape are given in Table 3. Any of them can be used as the diagnostic parameters of looseness or “health indicators” of bolted joints not only in the sieving screens but in other vibrating machines too. Alarm levels can be clearly interpreted since the clearances always increase the amplitudes of vibrations.

### 5.2. Verification of Robustness to Impulsive Noise

Additionally, the authors checked if the second natural frequency would change after simulating the additive impulsive noise. Based on previous research, authors determined that in the real working conditions, the maximum range of impulsive noise impacts usually does not exceed the amplitude of the machine vibrations more than three times and comes from the material falling on the upper sieve. Three kinds of noises—Noise1, Noise2, and Noise3—have been used in the simulation. The are assumed to have alpha-stable distribution with parameters presented in Table 4. The alpha parameter is understood as the characteristic exponent, beta as the skewness, gamma as the scale parameter, and delta as the location parameter.

**Figure 28:**
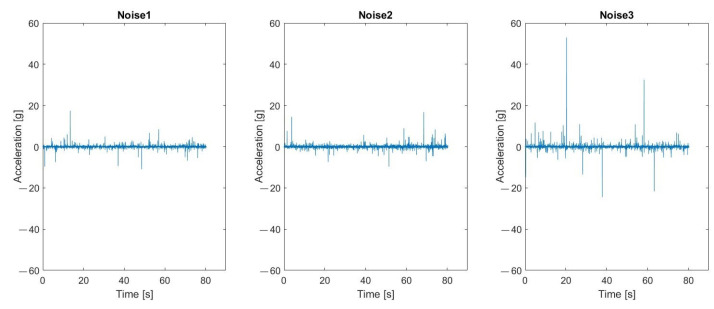
Visual representation of the generated noises.

**Figure 29:**
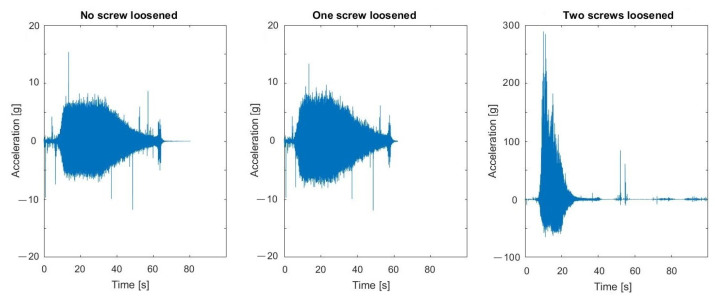
Visual representation of the original signals with added first of the generated noises.

Noises generated with these parameters can be seen in Figure 28 with an example of the original signal (coming from the Sensor A—on the sieve) convoluted with the first noise (cut to the moment of the machine being turned off) presented in Figure 29.

**Figure 30:**
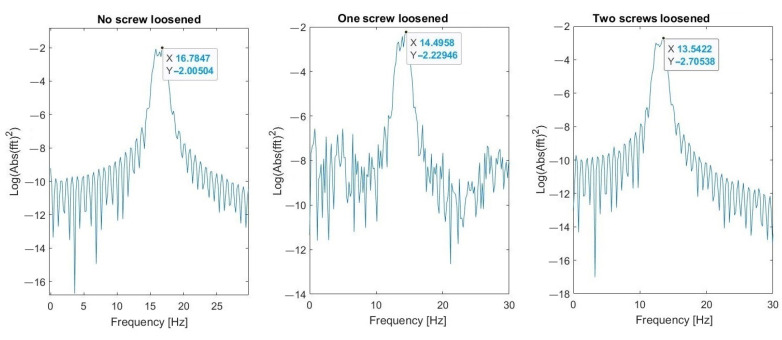
Second natural frequency after adding the first noise.

**Figure 31:**
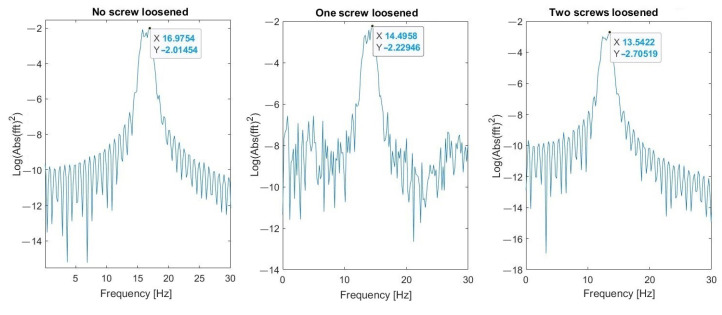
Second natural frequency after adding the second noise.

**Figure 32:**
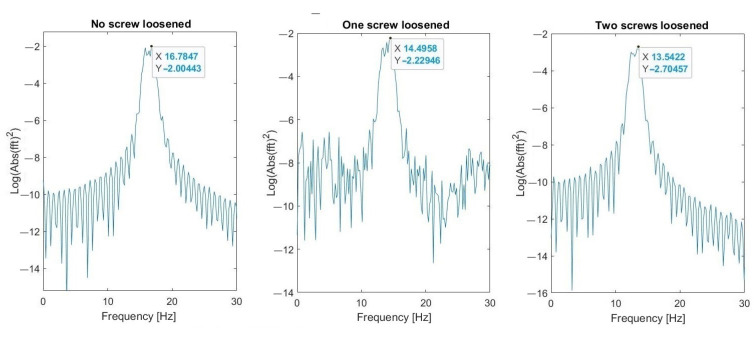
Second natural frequency after adding the third noise.

**Figure 33:**
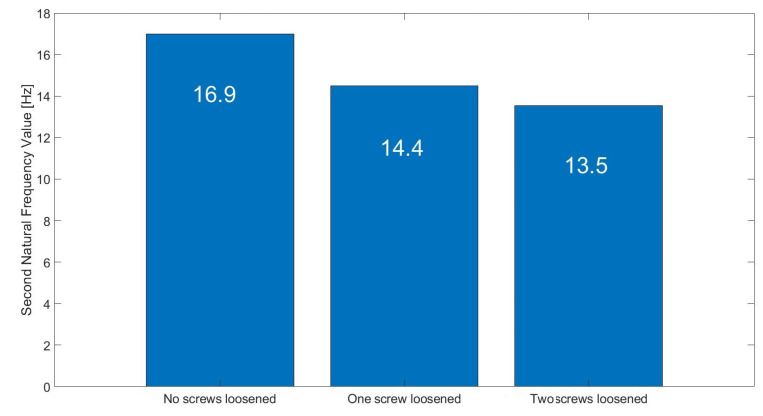
The calculated values of second natural mode frequency in three investigated cases.

Results visible in Figure 30, Figure 31 and Figure 32 show that the second natural frequency exhibits no significant changes after adding the impulsive noise, i.e., it is robust to external disturbances, which are always acting in the vibrating machines for bulk materials processing (compare values with Figure 24). The final dependence of the second mode natural frequency on bolt looseness is shown in Figure 33.

## 6. Discussion

The new approach to bolted joint diagnostics in the vibrating screens is developed based on the dynamical model and verified experimentally on the fully functional laboratory sieving screen.

No other scientific works on bolted joint diagnostics in the sieving screens were found during our research. The proposed in-the-market systems designed especially for the vibrating screens, e.g., CONiQ (Shenk) [83], SmartCheck (FAG) [84], ScreenWatch (Metso) [85], have no specific options for bolted joint diagnostics. Only a “Loose screen mesh” defect is noted in the Copperhead (SKF) system [86]. Hence, the developed method is an innovative solution for the industry.

The proposed approach, unlike other known studies, e.g., [11], considers the vibrating screen as a system with a changeable structure, which depends on bolted joints loosening. In other studies, the higher-order dynamical models including DEM and FEM simulations [19] were focused on the screen performance optimization along with a diagnostics of supporting springs [1] and bearings [34], while diagnostics of bolted joints was not covered.

If we are to compare the developed method with “smart” bolts or washers for loosening detection, our solution is more cost-efficient and convenient because it does not require additional time during screen maintenance for electronic component installation.

Vibration signals for diagnostic purposes can be recorded during the idle period of machine work to reduce the influence of impulsive noise from bulk material; however, this is usually not desirable in the continuous technological chains of large-scale enterprises. Therefore, to speed up and make the bolted joint diagnostics reliable, in distinction to the standard methods supposing detection of spectrum parameters changes at the kinematics-related frequencies, it is proposed to use a multi-body system of the natural mode frequencies, which are absolutely robust to impulsive noise, speed, and load variations. Identification of the first two natural modes frequencies can be accomplished by the calculation or a bump test conducted on the machine. Also, the signals during the system transition through the resonance are appropriate.

It was discovered, by experiments and on the model, that for certain combinations of design parameters of vibrating screens, the additional excitation (not accounted for at the development) can be generated by the higher (second, third) harmonics of the main frequency of inertial vibrators in case of their bearings’ deterioration. This fact is well-known in diagnostics but is firstly highlighted here as an initiating factor for bolted joint failures.

The gradual looseness of bolted joints appears as a result of the out-of-phase motion of the upper deck and the main body of the screen. The initial clearance induces a further increase in impact amplitude in the non-linear mechanical system. In conjunction with a standard deformation diagram (see Figure 11), this process can be described by the graph where maximum tensile stress in the bolts is dependent on a clearance gap size (see Figure 12). It can be applied in maintenance practice to visualize the limiting values of clearances in relation to required clamping force (torque) and bolted joint strength in different industrial machines.

The simulations on the 2-DOF dynamical model and experiments showed that the second mode frequency of vertical vibration can be a diagnostic parameter for bolted joint looseness detection. This frequency decreases with clearance development in the bolted joints due to their non-linear stiffness. The first mode frequency has no remarkable change with clearance development.

The damping factor of natural vibrations is identified as an additional diagnostics parameter, which decreases with clearance development in the bolted joints. The most appropriate periods for damping calculation are during the transient processes of the machine running out (stopping of vibrators) when it passes the internal resonances.

Phase space plots in coordinates of displacement and velocity provided a sensitive diagnostic method for bolt looseness. The advantage of these functions is in only one direction of vibration measurement. The proposed health indicators describing the shape of PSP trajectory, natural frequency, and damping are quite easily calculated in the time domain. They can be implemented in the even middle range of cost data collectors.

The most important data obtained in the test are quantitative relations of diagnostic parameters to the bolt loosening grades. The sensitivity by the second natural mode frequency is estimated at about 12.5 Hz/mm for initial loosening and about 4.5 Hz/mm for higher loosening. This regularity agrees with model simulations (see Figure 14). The damping ratio at the first natural mode is less sensitive, 11% and 12.5% for initial and higher bolt loosening, respectively, which agrees with simulations (see almost constant width of a first resonance peak in Figure 16). Although the laboratory screen had quite a different design as compared to the industrial screen, the proposed approach and selected diagnostic parameters proved to be similar to the model character and showed acceptable sensitivity, proving the applicability of our concept in practice. The different artificial impulsive (non-Gaussian) noise imposed on the measurement data obtained on the laboratory screen did not affect the results of the diagnostics. This is because external impacts cannot change the natural frequencies of the mechanical system.

Based on system modal analysis, the frequency response functions are constructed, which show that the system has anti-resonances. Keeping vibrators’ frequency close to a such region of *FRF*${}_{11}$ function will lead to excessive energy consumption and less productivity of the vibrating machine. The same effect produces bad condition of bolted joints due to vibrators’ energy leaking to the out-of-phase vibrations of the upper deck as the second mass and distortion of the designed trajectory of motion. Such dynamic effects are first considered in this research.

Vibrations at the second mode frequency and its higher harmonics can generate additional shock impacts with a wide-band spectrum on the upper deck, which intensifies the disintegration of cohesive fine-fractions materials with high humidity. However, it would be much more harmful to the bolted joints due to increasing the risk of their abrupt failures at the unpredicted moment of machine operation.

The loosened bolted joints will significantly disturb the designed trajectory (orbit) of the sieving screen motion and reduce its technological efficiency. The out-of-phase vibrations of the upper deck will cause the gradually increasing plastic deformations of bolts and finally lead to their breaking. If we assume the standard endurance limit of about $10^{6}$ cycles on the S-N curve and the frequency of the vibrator to be 15 Hz, then the expected time until bolt loosening and probable failure is about 18 h, if amplitudes of loading cycles are above the endurance limit of bolt material. This time span should be considered as a random value because of uncontrollable pre-tightening and material properties variation.

The higher yield stress of used steel supposes bigger amplitudes of impacts, which bolts can restrain without plastic deformation and irreversible elongation, which reduces tightening (tension) and finally produces a gap in the joint. On the other hand, usually, more strengthened steels have less impact toughness and more risk of brittle cracking under dynamic loading. Therefore, steel grade for bolts in the upper deck joints should be thoroughly selected to have simultaneously high yield stress, hence, higher endurance limit, and high impact toughness. Such contradictory properties can provide special heat treatment routes for alloy steels including the deep cryogenic treatment stage, which also eliminates internal stresses after heat treatment [87,88].

## 7. Conclusions

Based on the conducted research and vibrating screen simulations we have come to the following conclusions.

The detailed analysis of modal parameters allows considering the vertically vibrating screen structure as the 2-DOF system where the upper deck consisting of large section beams is considered as the second mass and its bolted joints as the intermediate stiffness with the main structure of the screen.

The total stiffness of these bolted joints has a non-linear (piece-wise linear) characteristic due to the unavoidable plastic deformation of bolt bodies under the action of severe impacts from the vertical components of excitation forces from unbalanced vibrators.

The gradual deterioration of bolts and appearing of clearances in the joints greatly increase the shock impacts produced by upper deck mass and further quick elongation of bolts within several working days. Therefore, their diagnostics and timely maintenance (tightening or replacement) are important for plant staff.

Using higher classes of bolts’ strength has not solved the problem because the main reason for that is a coincidence of the second mode frequencies of screen natural vibration with the main excitation force (15 Hz) and its higher harmonics (30 Hz, 45 Hz), which appears when shaft bearings and spring supports are in bad technical condition.

Such interrelation of failures in different units of industrial machines is well known in condition monitoring and explicitly undermined for the vibrating screen in current research.

At the design stage, to minimize the effect of out-of-phase vibrations in the sieving screens, which increase the energy consumption and risk of bolted joints failure, it is necessary to keep the anti-resonance frequency beyond of at least 5 Hz range from the rotation frequency of the inertial vibrators. During the screen operation, maintenance personnel should periodically check the wear of bearings in vibrators either manually (with dial micrometers or calibrated gauges) or by the signals of condition monitoring systems where it is reflected in the amplitudes of higher harmonics of rotation frequency.

A possible way to increase the maintenance interval on the existing screen is to introduce elastic pads on both sides of bolted joints. In this case, the stiffness tuning between masses allows shifting the frequency of the second natural mode from the frequency of the main excitation force and its higher harmonics.

In this research, only those bolts are considered, which are fastening the upper deck to the screen structure. The other bolts connecting side panels and reinforcement bars, which loosening is related to higher natural modes of the screen structure, are planned for analysis in our future research.

## Figures and Tables

**Table 1:** Maintenance periods for vibrating screen elements.

Nr	Action	Maintenance Period				
		50 h	Week	Month	Year	2 Years
1	Lubrication of vibrator bearings					
2	Control of sieve mounting					
3	Control of sieves and vibrators					
4	Control of springs					
5	Control of belt drives					
6	Inspection of sieve wear					
7	Inspection of drives					
8	Inspection of vibrators					

**Table 2:** Parameters of the dynamical model.

Parameter	Value	Units
Mass of screen body $m_{1}$	15,000	kg
Mass of upper deck $m_{2}$	5450	kg
Stiffness of supporting springs $k_{1}$	$0.56\times 10^{7}$	N/m
Stiffness of bolted joints $k_{2}$	$1.46\times 10^{8}$	N/m
Damping in supporting springs $b_{1}$	10	s${}^{-1}$
Damping in bolted joints $b_{2}$	10	s${}^{-1}$
Clearance in bolted joints Δ	0.0–$1.2\times 10^{-3}$	m

**Table 3:** PSP shape parameters of the signals ($\times 10^{-3}$) from Figure 27.

Parameters	Case (a)	Case (b)	Case (c)
$D_{min}$	−0.18	−0.19	−25.68
$D_{max}$	0.18	0.19	24.44
$V_{min}$	−24.60	−25.14	−79.57
$V_{max}$	24.10	23.97	77.99
$\Delta D=D_{max}-D_{min}$	0.36	0.38	50.12
$\Delta V=V_{max}-V_{min}$	48.70	49.11	157.56
ΔD×ΔV	17.51	18.82	7897.11

**Table 4:** Parameters of the generated alpha-stable noises.

Parameter	Noise1	Noise2	Noise3
Alpha	1.7	1.9	1.5
Beta	0.01	0.03	0.005
Gamma	0	0	0
Delta	0	0	0

## Data Availability

Data is available upon request to the corresponding author.
